# Dynamics based on analysis of public data for spreading of disease

**DOI:** 10.1038/s41598-021-91024-6

**Published:** 2021-06-09

**Authors:** Leonardo S. Lima

**Affiliations:** Federal Center for Technological Education of Minas Gerais, Belo Horizonte, MG 30510-000 Brazil

**Keywords:** Biophysics, Physics

## Abstract

The stochastic model for epidemic spreading of the novel coronavirus disease based on the data set supply by the public health agencies in countries as Brazil, United States and India is investigated. We perform a numerical analysis using the stochastic differential equation in Itô’s calculus for the estimating of novel cases daily, as well as analytical calculations solving the correspondent Fokker–Planck equation for the probability density distribution of novel cases, *P*(*N*(*t*), *t*). Our results display that the model based in the Itô’s diffusion fits well to the results due to uncertainty in the official data and to the number of tests realized in populations of each country.

## Introduction

The coronavirus disease (COVID-19) emerged in China in the end of 2019 year and since then it has spread reaching all countries in the globe. The quick spreading of the disease made the study of spreading of great importance for forecasting, control and prevention^1–5^. On the other hand, the modelling of natural phenomena up to end of nineteenth century can be viewed as the study of deterministic solutions of differential equations^6,7^. At that time thought that whether all initial data could only be collected, one would be able to predict the future with certainly. We now know this is not so since that the concept of chaos has arisen, in which even quite simple differential equation systems have the rather property of giving rise to unpredictable behavior^8,9^. A historical example is the Brownian motion or the motion viewed of pollen grains in suspension in the fluid, discovered by the British botanic Robert Brown who observed firstly the motion in 1827^8^. It was not quickly solved, and a satisfactory explanation did not come up to 1905 by Einstein. The same explanation was independently developed by Smoluchowski. The Einstein’s results were derived later by Langevin who writes in the form of the following differential equation $dx/dt=a(x,t)+b(x,t)\xi (t)$. *a*(*x*, *t*) and *b*(*x*, *t*) are known functions and $\xi (t)$ is a rapidly fluctuating random term. In general, an idealised mathematical formulation of the concept of a highly irregular function is that, for $t\ne t'$, *x*(*t*) and $\xi (t')$ are statistically independent^8,9^. In addition, we require $\langle \xi (t)\rangle =0$, since any nonzero mean can be absorbed into the definition of *a*(*x*, *t*), and thus require that $\langle \xi (t)\langle \xi (t')\rangle =\Gamma \delta (t-t')$. $\Gamma$ is the amplitude of the noise that satisfies the requirement of no correlation at different times and has the rather result that $\xi (t)$ has infinite variance. The Langevin equation was so the first type of a stochastic differential equation. An derivation in a foot mathematically more rigorous of the Langevin equation was performed later by the mathematicians Itô and Stratonovich^9,10^.

It is known that the logistic model of population growing can be used as model for some infectious diseases as the smallpox that are spread largely by individuals who can transmit the disease but who exhibit no overt symptoms^7^. The logistic model may be modified so that unbounded growth does not occur. The simplest way to do this is to introduce a factor which will have the effect of making the variation rate of population growth to be $d\wp /dt$ negative when *z* is large. Where the model is given in form $d\wp /dt=-\alpha \wp (1-\nu \wp )(1-\eta \wp )$, where $\alpha$ and $\eta$ are parameters and $\alpha >0$, $0<\eta <\nu$. Moreover, we can allow some randomness in these parameters with aim to obtain a more realistic model of real situation^8–12^. In a general way, if the size of the population at time *t* is $\wp (t)$ and $\varpi (t)$, being the relative rate of growth at time *t*, we can have $\varpi (t)$ subjects to some randomness due to environmental effects, so that $\varpi (t)=\wp (t)+\xi (t)$. Where the first term $\wp (t)$ is deterministic while the second term $\xi (t)$ reflects to the environmental randomness effect. Particularly, whether $\xi (t)$ presents the properties $\langle \xi (t)\rangle =0$ and $\langle \xi (t)\xi (t')\rangle =\Gamma \delta (t-t')$ with $\Gamma$ constant being the amplitude of the white noise.

In this paper, we investigate the Itô’s stochastic diffusion model as possible model for the spreading of the COVID-19. The use of this type of analysis has been employed in the study of the behavior of time series for the price dynamics in Refs.^13–16^. The stochastic analysis was employed for statistical inference in a stochastic epidemic model for the Ebola virus in Democratic Republic of Congo in 1995, using the Monte Carlo method which is used to explore the distribution of the parameters in Ref.^17^. FFurthermore, a discrete-time stochastic epidemic model, with distribution binomial to study the transmission rate of the COVID-19 has been studied in Ref.^18^. Where the parameters of the model were estimated fitting to the novel data and using simulations, where one showed a trending of declining in the total number confirmed cases. In Ref. Cite Kaustuv, a stochastic model for health care impact of the epidemic of novel coronavirus in India has been studied in using Monte Carlo simulation, where the hospitalization, intensive care unit requirements and deaths were modeled and the impact of social measures distance and lockdown on checking of the epidemic was estimated. In Ref.^19^ was developed a stochastic approach described by the master equation and transition rates for the infection process. Here, we aim to use the stochastic nonlinear differential equation in Itô’s calculus for modeling of the dynamic of novel cases daily as well as the cumulative cases number since the beginning of pandemic until today. We use different statistical tests to give a future estimating of novel cases and behavior of the curve of the spreading of disease by solving the stochastic differential equation and using the probability density *P*(*N*, *t*), obtained analytically from solution of the Fokker–Planck equation. Due to large uncertainly in the official data about the real number of novel cases generated by the low number of tests realized in countries as Brazil, where the effect of randomness in the official data is modelled by the random term in the stochastic differential equation, being the use of this analysis hence, largely adequate to treat the spreading of coronavirus disease. As far as we know, there are none work that employs the modeling through nonlinear stochastic differential equations of Itô for the modelling of the spreading of the coronavirus. The plan of this paper is the following. In section "Phenomenological model for dynamics of the cumulative cases number and novel cases", we describe the stochastic model. In section "Nonlinear Fokker–Planck equation", we present the numerical results by stochastic differential equation. In section "Analytical results by Fokker–Planck equation", we perform an analytical calculations, solving the correspondent Fokker–Planck equation. In section "Conclusions", we present our final remarks.

## Phenomenological model for dynamics of the cumulative cases number and novel cases

### Model for dynamics of the cumulative cases number

**Figure 1:**
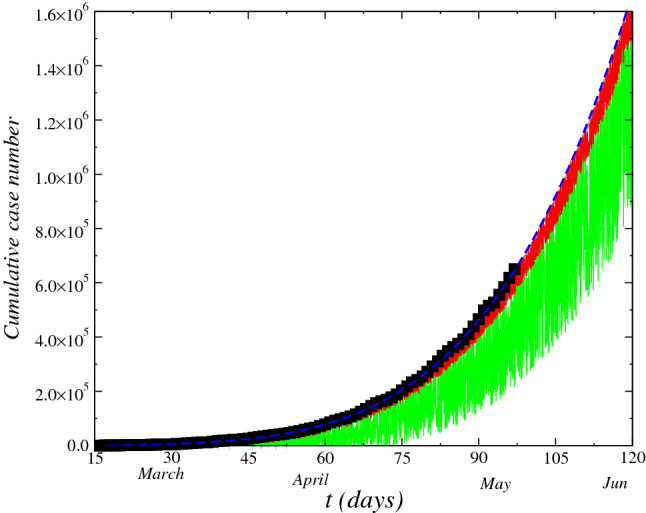
Effect of the stochastic term on cumulative total case number of COVID-19, ${\mathscr {P}}(t)$, in Brazil. We perform the calculations for different values of strength of randomness $\beta _0$ in model Eq. (1): $\beta _0=0.0$ (dashed blue-line), $\beta _0=3.0\times 10^{-6}$ (red-line) and $\beta _0=5.0\times 10^{-6}$ (green-line).

Based on the logistic growth with a threshold, considering that the size of the population of infected at time *t* is ${\mathscr {P}}(t)$ and the novel cases number daily is *N*(*t*),we can have the growth of ${\mathscr {P}}$ and *N* as a function of *t*, subject to environmental random effects so that $d{\mathscr {P}}(t)/dt = f(t)+A({\mathscr {P}}(t),t)+\xi (t)$. Where the first term is deterministic while the second term $\xi (t)$ reflects to the environmental randomness effect. In addition, $\xi (t)$ presents the properties $\langle \xi (t)\rangle =0$ and $\langle \xi (t)\xi (t')\rangle =\Gamma \delta (t-t')$ with the $\Gamma$ constant being the amplitude of the noise. The behavior of the cumulative total cases number ${\mathscr {P}}(t)$ of infected by coronavirus registered in the Brazil as function of time (days) from $15^{th}$ March, 2020 is displayed in Fig. 1. The data were registered within the period. The non differential points are due to uncertainty in the official data and to population isolation conditions. Hence, for modeling of this behavior, we has added a randomness together with nonlinear terms in Eq. (1) with aim to simulate the effect of this uncertainty.

1 $$\begin{aligned} d{\mathscr {P}}(t)=(f(t)+A({\mathscr {P}}(t),t))dt+B({\mathscr {P}}(t),t)\circ dW \end{aligned}$$

where *f*(*t*) is a polynomial of *n* degree obtained from least squares fit to set of data. Furthermore, we have a deterministic term $A({\mathscr {P}}(t),t)$ in the equation above, given by the logistic model with threshold, $A({\mathscr {P}}(t),t)=\alpha {\mathscr {P}}(t)(1-\nu {\mathscr {P}}(t))$ and the randomness term $B({\mathscr {P}}(t),t)=\beta _0t^3$ with the dependence of *t* of this term implying in a multiplicative white noise in Eq. (1). We perform the calculations for different values of $\beta _0$ constant which is the intensity of randomness, ( generated by low test number and to environmental conditions). We obtain a strong oscillation of the curve with the increasing of $\beta _0$ indicating thus, a trend of increase of growing of the uncertainly of the cumulative total case number. The data are considered from 15-March up to now.

### Model for dynamics of novel cases daily

The model for spread of novel cases of COVID-19 in each day is given by the modified logistic model of growth with threshold with the addition of randomness term given by the following stochastic differential equation (SDE) of Itô

2 $$\begin{aligned} dN(t)=h+\left[ g(t)-\alpha N(t)(1-\nu N(t))(1-\eta N(t))\right] dt+\beta (t) d{\mathscr {W}}. \end{aligned}$$

where $\alpha$, $\nu$ and $\eta$ parameters comes from the logistic model with threshold for spreading of the smallpox with the values $\alpha =\nu =0.125$ used by Bernoulli. Thus, the range of $\eta$ employed in calculations is within the interval $0<\eta <\nu$. *g*(*t*) is another fit least squares to the set data within the range considered. The deterministic part *g*(*t*), can adjust to the reported novel cases in each day *t* using a *n* degree ($n\le 4$) polynomial. The constant *h* is introduced due to the assumption of a constant rate when the novel cases number *N* is large, being this assumption reasonable in this limit. However, it becomes less reasonable when *N* is small. Furthermore, ${\mathscr {W}}(t)$ is the Wiener process or Brownian motion. Thus, we choose a multiplicative white noise of the form $\beta _0t^n$, where $\beta _0$ is an arbitrary constant. The above equation can also describe a particle in Brownian motion under action of a nonlinear potential^20^. Where the dissipating force is given by $-\alpha dN/dt$ (represented by the drift term in the Langevin equation) and the white noise term $\xi (t)$ if relates to the Wiener process as ${\mathscr {W}}(t)$ by ${\mathscr {W}}(t) = \int _{t_0}^{t}\xi (t')dt'$; $\langle \xi (t)\rangle = 0$; $\langle \xi (t)\xi (t')\rangle =\Gamma \delta (t-t')$.

**Figure 2:**
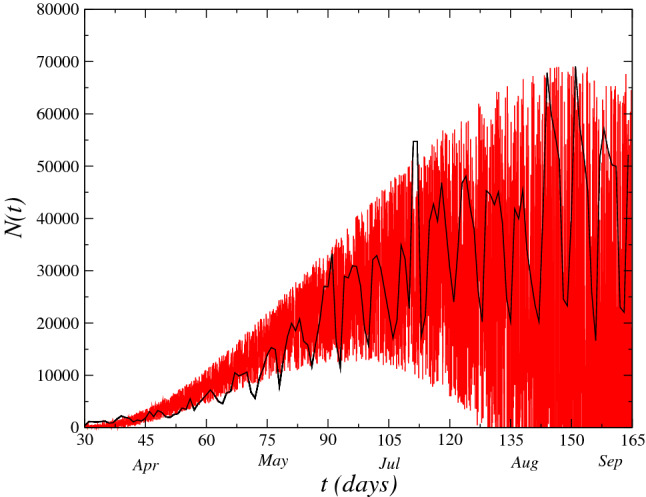
(Left) Dynamics of novel cases *N*(*t*) in Brazil. The zigzag behavior in the results is reflected by the stochastic term in Eq. (2). We plot the time series of the model Eq. (2) for $\alpha =\nu =0.125$, $\eta =0.0625$ and noise amplitude $\Gamma =1$ as well as $\beta (t)=3.0\times 10^{-6}t^3$.

**Figure 3:**
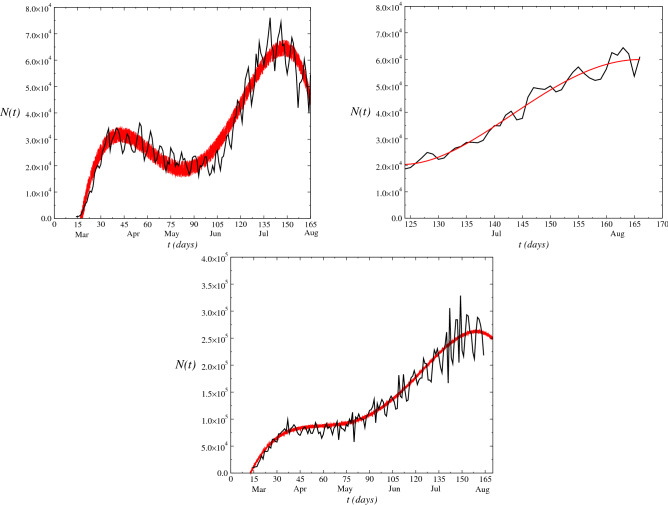
Dynamics of novel cases *N*(*t*) in United Sates (left), India (Center) and all world (right) and the correspondent time series obtained for each model Eq. (2). The data were obtained for values $\alpha =\nu =0.125$, $\eta =0.0625$, noise amplitude $\Gamma =1$ and $\beta =2.0\times 10^{6}$.

The dynamics of novel cases, *N*(*t*), of infected by COVID-19 registered in the Brazil as a function of time (days) registered in the period from 15th March, 2020 up to 12th August is displayed in Fig. 2. We obtain the time series of the model Eq. (2). From fit of least squares to the set of data supplied of the Brazilian agencies, we obtain the fit *g*(*t*) given by $g(t)= 8191.2 - 620.92 t + 12.63 t^2 - 0.05 t^3$. The zigzag behavior in the range of large *t* values reflects in an increase of the uncertainty in the data due to the low-number of test performed in the population. Consequently, for modeling of this behavior, we add the randomness term in the logistic model for growing of infectious disease as given by Eq. (2) with the aim to simulate the effect of this uncertainty. Although the model was based on Brazilian data only, it can be applied to the data of other countries as well. Since *g*(*t*) is the adjustments to the set of data of COVID-19 of each country where the uncertainty after a time *t* large becomes larger generating so, an increase of distance between the real data. In Fig. 3, we show the time evolution of the novel cases *N*(*t*) for some countries as United States and India. The range of data considered is up to August $12^{th}$. The set of data of the India presents a stronger oscillation into the range displayed in the figure, where in the beginning of the pandemic the oscillation was small (do not displayed in the figure). Therefore, the effect of the stochastic term is to span the fluctuations of the data (as displayed in Figs. 2 and 3). These daily fluctuations with a weekly cycle in the number of COVID cases reported in many countries which is mainly due to diagnostic and data reporting practices^21^. We obtain the time series of the model Eq. (2) in each case using the values $\alpha =\nu =0.125$, $\eta =0.0625$ and considering the noise amplitude $\Gamma =1$. Furthermore, we use in this case $\beta (t)=\beta _0$ (additive white) where: $\beta _0=2.0\times 10^{6}$.

### Numerical results

We perform the simulation of the model Eq. (2) for $\beta {\mathscr {W}}(t)$, whose standard deviation is given by $\sigma _{\mathscr {W}}=\sqrt{\Delta t}$. We write the Wiener increment as $\beta d{\mathscr {W}}(t)\sim \sqrt{dt} \beta R_G$, where $R_G$ is an aleatory generator number with Gaussian distribution of mean zero and variance $\sigma _{{\mathscr {W}}}^2=1$. In Fig. 2, we plot the time series of the model Eq. (2) for $\beta (t)=3.0\times 10^{-6}t^3$. For all case analyzed, we have the time series of novel cases oscillating quickly as displayed in the figure.

**Figure 4:**
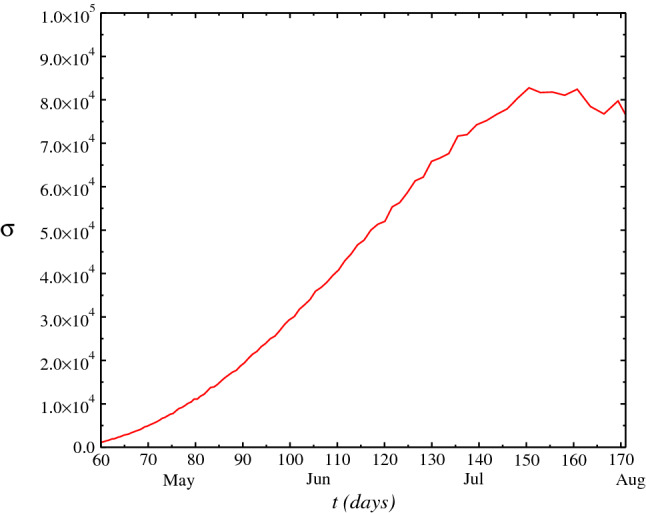
Behavior of the half-width of the distribution as a function of *t*, $\sigma (t)$. The half-width gives an expectation of new cases in each day *t*.

**Figure 5:**
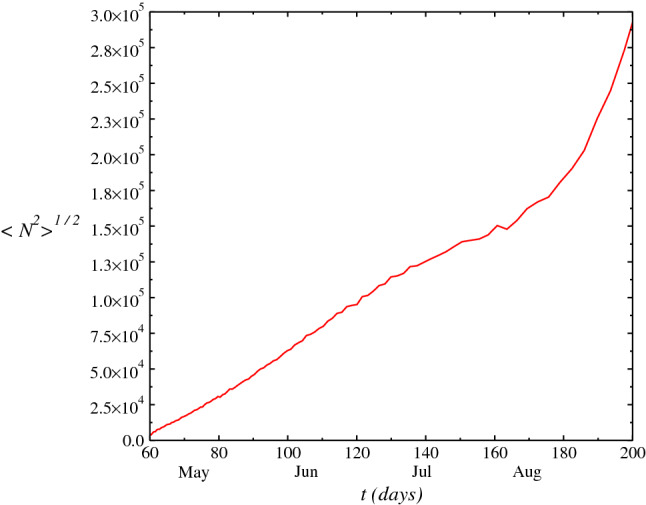
Mean square deviation root $\sqrt{\langle N^2\rangle }$ analogous to the mean-square root deviation of a particle in Brownian motion.

**Figure 6:**
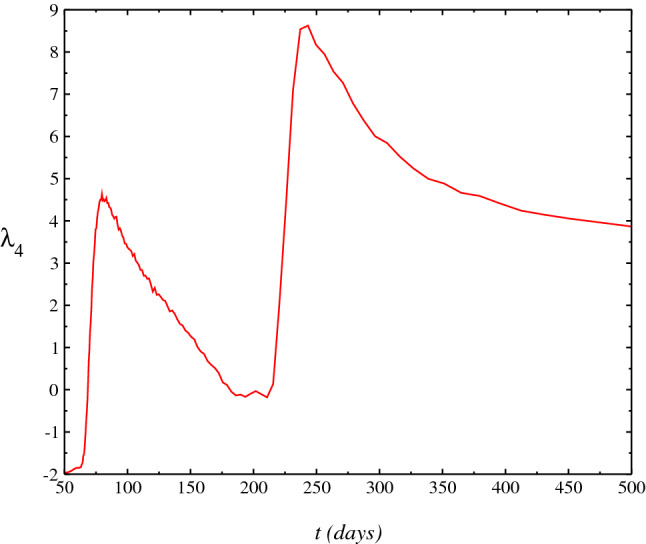
Behaviour of the kurtosis as a function of *t* $\lambda _4(t)$. The range of negative values gives an estimating of the shape of distribution which becomes nearest of a Gaussian for $\lambda _4=0$ at range of large *t* values since the firsts cases reported in the Brazil on $15^{th}$ March 2020.

**Figure 7:**
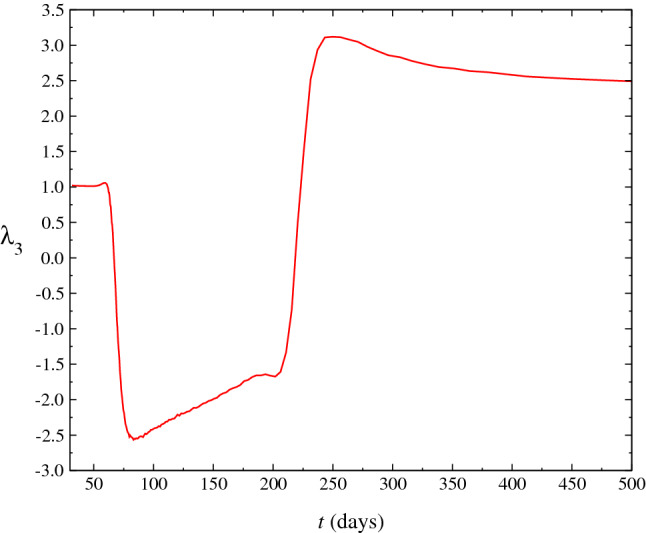
Behaviour of the skewness as a function of *t* $\lambda _3(t)$. The negative (positive) values obtained give an estimating of the shape of distribution that becomes nearest of a Gaussian when $\lambda _3\rightarrow 0$ at range of large *t* values since the firsts cases registered in Brazil on 15th March 2020.

In Fig. 4, we plot the half-width of the probability density *P*(*N*, *t*) as function of time *t*, $\sigma (t)$. We calculate the variance of the distribution where the standard deviation gives an estimating of novel cases in each day. The results adjust to the official data of ministry of healthy within range considered. The difference to the real data may be due to approach used. A treatment considering a nonwhite noise approach which approaches a delta-correlated noise may give a better adjusts to the real data. Anyway, it seems to exists a large probability of increase in the novel cases number within the next weeks up to a plateaus. In Fig. 5, we plot the mean-square deviation root $\sqrt{\langle N^2\rangle } =\sqrt{2\beta (t)^2 t}$ as function of *t*, with aim to calculate the analogous of the mean square displacement root $\sqrt{\langle x^2\rangle }$ that a particle experiences on the average or, the square root of the arithmetic mean of the square displacement. This reflects on behavior of a particle in Brownian motion in the fluid, where Einstein and Smoluchowski (on independent way) have derived the Einstein-Smoluchowski law. This law is a manifestation of the fluctuation-dissipation theorem and gives the mean-square deviation of the motion of a particle in suspension in a fluid: $\langle x^2\rangle \propto T$, where *T* is the temperature of the fluid and $x^2$ is the mean quadratic displacement. Consequently, we must have an analogous relation for the mean-square deviation of the novel cases $\langle N^2\rangle$.

We introduce the $n^{\mathrm {th}}$ order moments $\mu _n=\langle (x-m_1)^n\rangle$ about the mean or central moments, where one has the following relations: $c_1=\mu _1$, $c_2=\mu _2$, $c_3=\mu _3$, $c_4=\mu _4-3\mu _{2}^2$. Normalized measures often used, indicating a deviation from a Gaussian, are the kurtosis, $\lambda _4$ defined as $\lambda _4=\mu _4/\sigma ^4-3$ and the skewness, $\lambda _3$. In Fig. 6, we show the behavior of the kurtosis (excess), $\lambda _4(t)$. $\lambda _4(t)$ is numerically calculated solving the Eq. (29). Moreover, the kurtosis if relates to the deviation of the tail of the distribution, as compared to Gaussian $P(N,t)=\left( 1/\sqrt{4\pi \beta t}\right) e^{-N^2/4\beta t}$, whose solution would correspond to the Eq. (2) and (29)) for the cases $\nu =\eta =0$ and $g(t)\equiv 0$. The range of negative values obtained for the kurtosis indicates that the shape of the distribution is near to the Wigner semicircle^11^. Furthermore, at range of small *t* values, where the kurtosis is nearest to zero, we have that the distribution is nearest of a Gaussian distribution ($\lambda _4=0$). In Fig. 7, we perform another statistical test: the skewness, $\lambda _3(t)=c_3/c_2^{3/2}$. $\lambda _3(t)$ gives a measure of the degree of asymmetry of the distribution. When skewness is zero, we have a perfect Gaussian. Thus from results showed in the Figure, we find a large asymmetry in the distribution that must not obey hence, to the Gaussian distribution.

## Nonlinear Fokker–Planck equation

Corresponding to the models Eqs. (1) and (2), we can derivative the nonlinear Fokker–Planck equations for the probability density of the cumulative cases number and cases novel daily $P({\mathscr {P}},t)$ and *P*(*N*, *t*), respectively^8^.

### Nonlinear Fokker–Planck equation within the Itô prescription

In a general way, for a nonlinear Fokker–Planck equation given by

3 $$\begin{aligned} \frac{\partial P(x,t)}{\partial t}=D\frac{\partial ^2\left\{ \left[ P(x,t)\right] ^{2-q}\right\} }{\partial x^2}-\frac{\partial \left[ P(x,t)K(x,t)\right] }{\partial x}, \end{aligned}$$

where this equation is proposed in Ref.^22^. Here, we take the stochastic variable *X* be $X={\mathscr {P}}$ and *N*, in the models Eqs. (1) and (2), respectively. We obtain that the Eq. (3) is equivalent to the Itô stochastic differential equation

4 $$\begin{aligned} dX=A(X(t),t)dt+\phi (X(t),t)\circ dW, \end{aligned}$$

where $A(X(t),t)=K(X(t),t)$ and $\phi (X(t),t)=\left[ P(X(t),t)\right] ^\frac{1-q}{2}$. *X*(*t*) is a stochastic process defined on probability space $\Omega$. Being the triple $\left( \Omega ,{\mathscr {F}},P\right)$ a probability space, where ${\mathscr {F}}$ is a $\sigma$-algebra and *P* a probability measure. That is, a function that to every set $A\in {\mathscr {F}}$ assigns a number in range $\left[ 0,1\right]$, where $P(\Omega )=1$, $P(\emptyset )=0$ and

5 $$\begin{aligned} P\left( \bigcup _{n=1}^{\infty }A_n\right) =\sum _{n=1}^{\infty }P(A_n). \end{aligned}$$

The random variable *X*, defined on $\Omega$ with the property that for every Borel subset *B* of ${\mathbb {R}}$, the subset of $\Omega$ given by $\left\{ X\in B\right\} =\{\omega \in \Omega ;X(\omega )\in B\}$ is in the $\sigma$-algebra ${\mathscr {F}}$. Moreover, for *P*(*X*(*t*), *t*), given *X* a random variable on a probability space $\left( \Omega ,{\mathscr {F}},P\right)$, *P* is the probability measure of *X*. $\mu _X$ assigns to each Borel subset *B* of ${\mathbb {R}}$ the mass $\mu _X(B)=P\{X\in B\}$^9^.

The Itô integral for $\phi$ is given by

6 $$\begin{aligned} \int \phi (X(t),t)dW(t) =\hbox {ms}-\lim _{n\rightarrow \infty }\left\{ \sum _{j=1}^{n}\phi (t_{i-1}) \left[ W(t_i)-W(t_{i-1})\right] \right\} , \end{aligned}$$

where ms$-\lim$ means square limit. *W*(*t*) is a Markovian process, presenting normal distribution, satisfying the conditions $\langle \xi (t)\rangle =0$ and $\langle \xi (t)\xi (t')\rangle =\delta (t-t')$.

We can write the Eq. (3) as

7 $$\begin{aligned} \frac{\partial P(x,t)}{\partial t}=D\frac{\partial ^2\left\{ P(x,t)\left[ P(x,t)\right] ^{1-q}\right\} }{\partial x^2}-\frac{\partial \left[ P(x,t)K(x,t)\right] }{\partial x}, \end{aligned}$$

where we define $A(x,t)=K(x,t)$ and $\phi (X(t),t)=\left[ P(X(t),t)\right] ^\frac{1-q}{2}$ to obtain the correspondent Itô stochastic differential equation

8 $$\begin{aligned} dX=K(X(t),t)dt+\left[ P(X(t),t)\right] ^{\frac{1-q}{2}}\circ dW, \end{aligned}$$

which is the Eq. (4). The solution for *X*(*t*) using the Stratonovich integral is given by

9 $$\begin{aligned} X(t)=X(0)+\int _0^tK(s,X_s)ds+\int _0^t\phi (s,X_s)dW_s. \end{aligned}$$

This implies that *X*(*t*) is the solution of the following modified Itô equation

10 $$\begin{aligned} X(t)=X(0)+\int _0^tK(s,X_s)ds \end{aligned}$$

where $\phi '$ denotes the derivative of $\phi (x,t)$ with respect to *x*. Therefore, the Eq. (3) in Itô calculus is different of the Stratonovich interpretation.

From the Feynman-Kac theorem, let *h*(*x*) be a Borel-measurable function. Fix $T>0$, and let $t\in [0,T]$ be given, we define the function

11 $$\begin{aligned} g(x,t)=E^{t,x}h(X(T)), \end{aligned}$$

where

12 $$\begin{aligned} E|g(X)|=\int _{-\infty }^{\infty }|g(x)|P(x)dx. \end{aligned}$$

Furthermore, we assume $E^{t,x}|h(X(T))|<\infty$ for all *t* and *x*. Then *g*(*x*, *t*) satisfies the partial differential equation

13 $$\begin{aligned} \frac{\partial g(x,t)}{\partial t}+K(x,t)\frac{\partial g(x,t)}{\partial x}+\frac{1}{2}\phi ^2(x,t)\frac{\partial ^2g(x,t)}{\partial x^2}=0 \end{aligned}$$

with the terminal condition $g(x,t)=h(x)$ for all *x*, where we assume that the stochastic process *g*(*X*(*t*), *t*), $0\le t\le T$ is a martingale^9^.

From Eq. (4), we obtain the time development of an arbitrary *f*(*X*(*t*)) using the Itô formula^8^

14 $$\begin{aligned} f\left[ X(t)+dX(t)\right] -f\left[ X(t)\right] =\frac{\partial f}{\partial x}\bigg [K(X(t),t)dt +\left[ P(X(t),t)\right] ^\frac{1-q}{2}\circ dW\bigg ] +\frac{1}{2}\frac{\partial ^2f}{\partial x^2}\left\{ \left[ P(X(t),t) \right] ^\frac{1-q}{2}\right\} +{\mathscr {O}}(\cdot \cdot \cdot ). \end{aligned}$$

Taking the average of both sides in the equation above, we obtain

15 $$\begin{aligned} \left\langle \frac{df}{dt}\right\rangle =\left\langle \left[ \frac{df}{dx}K(X(t),t) +\frac{1}{2}\frac{\partial ^2f}{\partial x^2}\left\{ \left[ P(X(t),t)\right] ^\frac{1-q}{2}\right\} \right] \right\rangle +\frac{d}{d t}\left\langle \left\{ \left[ P(X(t),t)\right] ^\frac{1-q}{2}\right\} \right\rangle \end{aligned}$$

and using

16 $$\begin{aligned}&\frac{\langle f(X(t)\rangle }{dt}=\frac{d}{dt}\int _{-\infty }^{\infty }dx f(x)P(x,t)\nonumber \\&\quad =\int _{-\infty }^{\infty }dx f(x)\frac{\partial }{\partial t}\left[ P(x,t)\right] =\int _{-\infty }^{\infty }\frac{\partial f}{\partial x}K(x,t)P(x,t)dx+\frac{1}{2}\int _{-\infty }^{\infty }\frac{\partial ^2f}{\partial x^2}\left\{ \left[ P(x,t)\right] ^\frac{1-q}{2}\right\} P(x,t)dx +\int _{-\infty }^{\infty }dxf(x)\frac{\partial P}{\partial t}. \end{aligned}$$

We integrate by parts and discard surface terms to obtain

17 $$\begin{aligned} \int _{-\infty }^{\infty }dx f(x)\frac{\partial }{\partial t}\left[ P(x,t)\right] =\int _{-\infty }^{\infty }f(x)\frac{\partial }{\partial x}\left[ K(x,t)P(x,t)\right] dx +\frac{1}{2}\int _{-\infty }^{\infty }f(x)\frac{\partial ^2}{\partial x^2} \left[ \left\{ \left[ P(x,t)\right] ^\frac{1-q}{2}\right\} P(x,t)\right] dx. \end{aligned}$$

and hence

18 $$\begin{aligned} \frac{\partial }{\partial t}P(x,t)=-\frac{\partial }{\partial x}\left[ K(x,t)P(x,t)\right] +\frac{1}{2}\frac{\partial ^2}{\partial x^2}\left\{ \left\{ \left[ P(x,t)\right] ^\frac{1-q}{2}\right\} ^2P(x,t)\right\} . \end{aligned}$$

Thus, we have a complete equivalence between the diffusion process defined by a drift coefficient *K*(*x*, *t*) and a diffusion coefficient given as $\phi (x,t)=\left[ P(x,t)\right] ^{1-q}$, in which the diffusion process can be locally approximated by an Itô stochastic differential equation.

Corresponding to Stratonovich stochastic differential equation

19 $$\begin{aligned} (S)dX=K^s(X(t),t)dt+\left[ P(X(t),t)\right] ^{\frac{1-q}{2}}dW, \end{aligned}$$

with $K^s=K-\frac{1}{2}\phi \partial _x\phi$ and using the correspondence between the Itô stochastic differential equation and Fokker–Planck equation, we have a equivalent Fokker–Planck equation

20 $$\begin{aligned} \partial _t P=-\partial _x\left\{ K^sP\right\} +\frac{1}{2}\partial _x \left\{ \phi \partial _x\left[ \phi P\right] \right\} , \end{aligned}$$

which is known as Stratonovich form of the Fokker–Planck equation^8^. However, it is different from Eq. (3). Therefore, we have that the corresponding nonlinear Fokker–Planck equation into the Stratonovich prescription is different from nonlinear equation obtained into the Itô prescription, being the Itô stochastic differential equation more usually employed to make the connection with the Fokker–Planck equation. In spite of both definitions can be related by the choosing of *i* by $\tau _i=\alpha t_i+(1-\alpha )t_{i-1}$, $\alpha \in (0,1)$, they generate different definitions for the stochastic integral (Itô integral and Stratonovich integral respectively) and consequently to different stochastic differential equations. Even though the Itô stochastic differential equation is equivalent to an another Stratonovich equation however, with an additional term^8^.

### Existence and uniqueness

We can investigate the existence and uniqueness of solutions of the nonlinear differential equations utilizing the well-known existence and uniqueness theorem for stochastic differential equations^9^. Let $T>0$ and $K(x,t):[0,T]\times {\mathbb {R}}^n\rightarrow {\mathbb {R}}^n$, $\phi (x,t):[0,T]\times {\mathbb {R}}^n\rightarrow {\mathbb {R}}^{n\times m}$ be measurable functions satisfying

21 $$\begin{aligned} {} |K(x,t)|+|\phi (x,t)|\le C(1+|x|); x\in \mathbb {}{R}^n, t\in [0,T] \end{aligned}$$

for some constant *C* and such that

22 $$\begin{aligned} |b(x,t)-b(y,t)|+|(\phi (x,t)-\phi (y,t)|\le D|x-y|; x,y\in {\mathbb {R}}^n,\quad t\in [0,T] \end{aligned}$$

for some constant *D*. Let *Z* be a random variable that is independent on the $\sigma$-algebra ${\mathscr {F}}_{\infty }^{m}$ generated by $W_s(\cdot )$, $s\le 0$ and such that the expectation $E\left[ |Z|^2\right] <\infty$. Then the stochastic differential equation

23 $$\begin{aligned} dX=A(X(t),t)dt+\phi (X(t),t)dW,0\le t\le T,X_0=Z \end{aligned}$$

has a unique *t*-continuous solution $X_t(\omega )$ with the property that $X_t(\omega )$ is adapted to filtration ${\mathscr {F}}_t^Z$ generated by *Z* and $W_s(\cdot )$; $s\le t$

24 $$\begin{aligned} E\left[ \int _0^T|X_t|^2dt\right] <\infty . \end{aligned}$$

Even though in literature has been used the Stratonovich equation to make the connection with the Fokker–Planck equation^23^, we have used here the Itô’s stochastic differential equation (which is equivalent to a Stratonovich equation with an additional term) to make the connection with the Fokker–Planck equation being hence, different from prescription usually made.

## Analytical results by Fokker–Planck equation

We can perform an analytical analysis solving the correspondent Fokker–Planck equation to the stochastic differential equation Eq. (2). We start from the time development of an arbitrary function of the stochastic process *N*(*t*), *f*(*N*(*t*)). Using the Itô formula

25 $$\begin{aligned} f\left[ N(t)+dN(t)\right] -f\left[ N(t)\right] =\partial _Nf[N(t)]\left\{ \left[ g(t') -\alpha N(t')(1-\nu N(t'))(1-\eta N(t'))\right] dt +\beta d{\mathscr {W}}\right\} +\frac{\beta ^2}{2}\partial _N^2f[N(t)](d{\mathscr {W}})^2 , \end{aligned}$$

where the higher order terms have been discarded, and $(d{\mathscr {W}}(t))^2=dt$. Taking the average of both sides in the equation above, we find

26 $$\begin{aligned} \bigg \langle \frac{\partial f}{\partial t}\bigg \rangle =\bigg \langle \left[ \frac{\partial f}{\partial x}\left\{ \left[ g(t)-\alpha x(1-\nu x)(1-\eta x)\right] dt +\beta d{\mathscr {W}}\right\} +\frac{\beta ^2}{2}\frac{\partial ^2f}{\partial x^2}\right] \bigg \rangle . \end{aligned}$$

In following, using

27 $$\begin{aligned}&\frac{d}{dt}\langle f(N(t)) \rangle =\frac{d}{dt}\int _{-\infty }^{\infty }dx f(x)P(x,t)=\int _{-\infty }^{\infty }dx f(x)\frac{\partial }{\partial t}\left[ P(x,t)\right] =\int _{-\infty }^{\infty }\frac{\partial f}{\partial x}\left\{ \left[ g(t) -\alpha x(1-\nu x)(1-\eta x)\right] \right\} P(x,t)dx\nonumber \\&\quad +\frac{\beta ^2}{2}\int _{-\infty }^{\infty }\frac{\partial ^2f}{\partial x^2} P(x,t)dx, \end{aligned}$$

we integrate by parts and discard surface terms to find

28 $$\begin{aligned} \int _{-\infty }^{\infty }dx f(x)\frac{\partial }{\partial t}\left[ P(x,t)\right] =\int _{-\infty }^{\infty }f(x)\frac{\partial }{\partial x}\left\{ \left[ g(t)-\alpha x(1-\nu x)(1-\eta x)\right] P(x,t)\right\} dx+\frac{\beta ^2}{2}\int _{-\infty }^{\infty }f(x) \frac{\partial ^2}{\partial x^2}\left[ P(x,t)\right] dx. \end{aligned}$$

and hence

29 $$\begin{aligned} {} \frac{\partial }{\partial t}P(x,t)=-\frac{\partial }{\partial x}\left\{ \left[ g(t)-\alpha x(1-\nu x)(1-\eta x)\right] P(x,t)\right\} +\frac{\beta ^2}{2}\frac{\partial ^2}{\partial x^2}P(x,t). \end{aligned}$$

Taking the Fourier transform of the above equation, we can guarantee the normalization of the probability density where *P*(*x*) is well behaved. We take the boundaries at infinity as $\lim _{x\rightarrow \infty } P(x,t)=0$ and $\partial _x P(x)$ being reasonably well behaved. As $\lim _{x\rightarrow \infty }\partial _x P(x,t)=0$ so, a nonzero current of probability at infinity will usually require that the terms in the equation above will become infinite there^8^. We use the initial condition $P(x_0,0)=P_0$.

For solving the Fokker–Planck equation time independent we make the power series expansion $P(x,t)=\sum _{n=0}^{\infty }a_n(t)x^n$ to find

30 $$\begin{aligned}&\frac{\partial P}{\partial t} =\alpha (1-2\nu x)(1-\eta x)P -\left[ g(t)-\alpha x(1-\nu x)(1-\eta x)\right] \frac{\partial P}{\partial x} \nonumber \\&\quad +\frac{\beta ^2}{2}\frac{\partial ^2P}{\partial x^2} \sum _{n=0}^{\infty } \left( \frac{da_n}{dt}+g(t)(n+1)a_{n+1}\right) x^n=\left[ \alpha (1-2\nu x)(1-\eta x)\right] \sum _{n=0}^{\infty }a_nx^n +\alpha x(1-\nu x)(1-\eta x)\sum _{ n=0}^{\infty }na_nx^{n-1}\nonumber \\&\quad +\frac{\beta ^2}{2}\sum _{n=0}^{\infty }n(n-1)a_nx^{n-2}. \end{aligned}$$

We obtain the following recurrence relations

31 $$\begin{aligned} \alpha (1-2\nu x)(1-\eta x)\sum _{n=0}^{\infty } a_nx^n+\alpha x(1-\nu x)(1-\eta x)\sum _{n=1}^{\infty }(n+1)a_{n+1}x^n +\frac{\beta ^2}{2}\sum _{n=0}^{\infty }(n+1)(n+2)a_{n+2}x^n=k, \end{aligned}$$

where *k* is a separation constant. For $k=0$, we obtain other recurrence relations given by

32 $$\begin{aligned} a_{2}=-\frac{\alpha }{\beta ^2}a_{0},\quad a_{3}=-\frac{\alpha }{3\beta ^2}a_{1},\quad a_4=\frac{\alpha ^2}{6\beta ^4}a_{0},\quad a_5=\frac{\alpha ^2}{30\beta ^4}a_1. \end{aligned}$$

Additionally, we have

33 $$\begin{aligned} {} \frac{da_n(t)}{dt}+g(t)(n+1)a_{n+1}(t)=k. \end{aligned}$$

Therefore, we obtain *P*(*x*) in the form

34 $$\begin{aligned} P(x)=a_0\left( 1-\frac{\alpha }{\beta ^2}x^2+\frac{\alpha ^2}{\beta ^4}x^4 +\cdot \cdot \cdot \right) +a_1\left( x-\frac{\alpha }{3\beta ^2}x^3+\frac{\alpha ^2}{30\beta ^4}x^5\cdot \cdot \cdot \right) , \end{aligned}$$

where the constants $a_0$ and $a_1$ are determined by the initial conditions $P(0,0)=P_0$ and $\partial _xP(x,0)=0$ in $x=0$. We find $a_0=P_0$ and $a_1=0$. From the normalization condition, the second term in the density probability above must be zero and hence, all coefficients $a_1$ must cancel. Hence, we have

35 $$\begin{aligned} P(x,t)=P_0\left( 1-\frac{\alpha }{\beta ^2}x^2+\frac{\alpha ^2}{\beta ^4}x^4+\cdot \cdot \cdot \right) \end{aligned}$$

To ensure the normalization of the probability density, $P_0$ must be non zero only within the interval $-\varepsilon \le x\le \varepsilon$ and zero out it.

For $k\ne 0$, we have from Eq. (31) that $n=0$ and $a_2+\alpha a_0/\beta ^2=k$ and all $a_n$ higher are zero. Thus, we find from integration of the Eq. (33)

36 $$\begin{aligned} P(t)=\frac{\beta ^2}{\alpha }P_0 t[1-a_2(t)]+a_0(0)-\int _{0}^{t}g(t')a_1(t')dt'. \end{aligned}$$

We find the *n*th moments $m_n=\langle N^n\rangle =\int _{-\infty }^{\infty }N^nP(N,t)dN$, where the mean half-width of the probability distribution $\sigma =\sqrt{\langle N^2\rangle -\langle N\rangle ^2}$ gives an estimating of novel cases in the day *t*. From the solution of the Fokker–Planck equation, we find an analytical expression for the mean half-width of the distribution as function of time given by

37 $$\begin{aligned} \sigma (t)=\left[ \frac{\gamma }{\alpha }P_0t(1-a_2(t))+a_0(0) -\int _{0}^{t}g(t')a_1(t')dt'\right] \sqrt{\frac{[N(t)]^3}{3}-\frac{[N(t)]^4}{4}} \end{aligned}$$

In addition, we have ($n=0$) $da_0(t)/dt=0$ and so $a_0(t)=c$, $a_{1}=kf(t)/2$ and $P(t)=P_0t+c-k/2$, where we define $p_0=c-k/2$. Therefore, we have

38 $$\begin{aligned} {} \sigma (t)=\left( P_0t+p_0\right) \sqrt{\frac{[N(t)]^3}{3}-\frac{[N(t)]^4}{4}}. \end{aligned}$$

How the first cases were registered on March 15th ($t=15$), we obtain for the official results of the novel cases number: $p_0=-1.5$ an $P_0=0.1$, obtaining thus a concordance with the numerical results of the stochastic analysis. Thus, from probability density *P*(*N*, *t*), solution of the Fokker–Planck equation Eq. (29), we obtain analytically the results obtained before numerically by stochastic differential equation, Eq. (2), as the variance of the distribution, where the standard deviation gives an estimating of novel cases number daily.

## Conclusions

*In Brief*, we propose a stochastic model for the spread of the SARS-CoV-2 (COVID-19) in Brazil based in the nonlinear Itô’s diffusion model. Our results are compared with official data supplied by the Brazilian healthy agencies where due to large uncertainty in the results generated principally by the low number of tests made in the population and hence, to under reporting, generates a large uncertainly in the official results and consequently, the stochastic differential equation analysis becomes a more realistic model for growing of the total number of infected ${\mathscr {P}}$ and novel cases number *N*(*t*). We can use an approach beyond white noise limit to try to better describe the expansion dynamics what can be done in a future work. The model reported here is based on Brazilian data from March 15, 2020 which shows an upward trend in the coming weeks.

Solving the Fokker–Planck equation, Eq. (29), we obtain the probability density *P*(*N*, *t*) and an analytical expression for variance of the distribution, where the standard deviation gives an estimating of novel cases daily and hence, we re-obtain all results obtained before, numerically by stochastic differential equation. In addition, we obtain a correspondence between stochastic differential equation and nonlinear Fokker–Planck equation obtained in the framework of the non-additive statistical mechanics different of the connection made in the literature^23,24^.
